# Factors associated with decision time to seek care in the face of ischemic stroke^*^

**DOI:** 10.1590/1980-220X-REEUSP-2023-0075en

**Published:** 2023-08-25

**Authors:** Ludimila Santos Muniz, Mariana de Almeida Moraes, Rilary Silva Sales, Laís Silva Ribeiro, Brenda Silva Cunha, Pedro Antônio Pereira de Jesus, Elieusa e Silva Sampaio, Camila Rosalia Antunes Baccin, Carlos Antônio de Souza Teles, Fernanda Carneiro Mussi

**Affiliations:** 1 Empresa Brasileira de Serviços Hospitalares, Hospital Universitário Professor Edgard Santos, Salvador, BA, Brazil.; 2 Universidade Federal da Bahia, Faculdade de Enfermagem, Salvador, BA, Brazil.; 3 Universidade Federal da Bahia, Instituto de Ciências da Saúde, Salvador, BA, Brazil.; 4 Universidade Federal da Paraíba, Faculdade de Enfermagem, João Pessoa, PB, Brazil.; 5 Universidade Estadual de Feira de Santana, Instituto Gonçalo Moniz, Fiocruz, Salvador, BA, Brazil.

**Keywords:** Stroke, Time-to-Treatment, Emergency Medical Services, Nursing, Health Education, Health, Accidente Cerebrovascular, Tiempo de Tratamiento, Servicios Médicos de Emergencia, Enfermería, Educación en Salud, Salud, Acidente Vascular Cerebral, Tempo de Tratamento, Serviços Médicos de Emergência, Enfermagem, Educação em Saúde, Saúde

## Abstract

**Objective:**

To verify the association between sociodemographic, clinical, environmental, cognitive, and emotional factors and the decision time of people with ischemic stroke to seek a health service after the onset of symptoms or wake up stroke.

**Method:**

Cross-sectional study carried out from March to October 2019, with 304 patients, in a public hospital, a reference in neurology. Data obtained through interview and from medical records. Decision time was analyzed as a geometric mean. In the bivariate and multivariate analyses, linear regression was used and the Akaike Information Criterion was used to select the best model. Statistical significance of 5% was adopted.

**Results:**

The geometric mean of decision time was 0.30h (95% CI 0.23–0.39). The final model explained this time in 41%, showing an increase of 0.5 min for people with arterial hypertension; 10.8 min for those who waited for symptoms to improve; 1.4 min for those who were alone at the onset of symptoms; 3.9 min for those at home; 3.2 min for the ones at work; and 2.1 for those on the street/public space.

**Conclusion:**

The mean decision time for seeking a health service was high and influenced by clinical, environmental, cognitive, and emotional variables. The results guide nurses regarding health education.

## RESUMO

**Objetivo:**

Verificar a associação entre fatores sociodemográficos, clínicos, ambientais, cognitivos e emocionais e o tempo de decisão de pessoas com acidente vascular cerebral isquêmico para procurar um serviço de saúde após o início dos sintomas ou *Wake up stroke*

**Método:**

Estudo transversal realizado de março a outubro de 2019, com 304 pacientes, em hospital público, referência em neurologia. Dados obtidos por entrevista e no prontuário. O tempo de decisão foi analisado em média geométrica. Nas análises bivariadas e multivariada foi usada a regressão linear e o Critério de Informação de Akaike foi usado na seleção do melhor modelo. Adotou- se significância estatística de 5%

**Resultados:**

A média geométrica do tempo de decisão foi de 0,30h (IC 95% 0,23-0,39). O modelo final explicou em 41% esse tempo, mostrando aumento de 0,5 min para pessoas com hipertensão arterial; 10,8 min para quem esperou os sintomas melhorarem; 1,4 min para quem estava sozinho no início dos sintomas; 3,9 min para quem estava em casa; 3,2 min no trabalho e 2,1 na rua/via pública

**Conclusão:**

A média do tempo de decisão para procura de serviço de saúde foi alta e influenciada por variáveis clínicas, ambientais, cognitivas e emocionais. Os resultados orientam enfermeiros para a educação em saúde.

## RESUMEN

**Objetivo:**

Verificar la asociación entre factores sociodemográficos, clínicos, ambientales, cognitivos, y emocionales y el tiempo de decisión de las personas con accidente cerebrovascular isquémico para buscar un servicio de salud luego del inicio de los síntomas o *Wake up stroke*

**Método:**

Estudio transversal realizado de marzo a octubre de 2019, con 304 pacientes, en un hospital público, referencia en neurología. Datos obtenidos por entrevista y de la historia clínica. El tiempo de decisión se analizó como una media geométrica. En los análisis bivariados y multivariados se utilizó la regresión lineal y el Criterio de Información de Akaike para seleccionar el mejor modelo. Se adoptó una significancia estadística del 5%

**Resultados:**

La media geométrica del tiempo de decisión fue de 0,30h (IC 95% 0,23-0,39). El modelo final explicó este tiempo en un 41%, mostrando un aumento de 0,5 min para las personas con hipertensión arterial, 10,8 min para las que esperaron a que los síntomas mejoraran, 1,4 min para las que estaban solas al inicio de los síntomas, 3, 9 min para las que estaban en casa, 3,2 min para los que estaban en el trabajo y 2,1 en la calle/vía pública

**Conclusión:**

El tiempo promedio de decisión para buscar un servicio de salud fue alto e influenciado por variables clínicas, ambientales, cognitivas y emocionales. Los resultados orientan a los enfermeros hacia la educación en salud.

## INTRODUCTION

Strokes affect 17 million people worldwide annually, generating about six million annual deaths. The global burden of stroke remains rising, resulting in more than 100 million years of life lost^(1)^.

Stroke is the leading cause of permanent disability^(2)^, resulting in neurological deficits that are difficult to be restored, affecting survivors, family members, friends, and communities. Currently, about 80 million people are stroke survivors and experience limitations. At least two-thirds of survivors remain with some degree of disability and become dependent^(3)^.

In recent decades, the use of thrombolytic therapy in ischemic stroke (IS) and the care of victims in Stroke Units are among the most effective measures to reduce mortality and functional disability^(4)^. Although the thrombolytics are potentially effective agents, their effectiveness is related to early administration. At level 1A evidence, benefits are achieved up to four hours and thirty minutes after the onset of symptoms^(5)^. A delay of 15 minutes in care reduces a month of healthy life and every minute gained in speed in care yields 1.8 days of healthy life^(2)^.

Despite the established benefits of thrombolysis in stroke, international studies have highlighted the delay in the arrival of victims to health services, implying in the loss of the therapeutic window^(6,7)^. The reasons identified included lack of knowledge and misjudgment of symptoms and signs, lack of understanding of the severity of the event, hope for improvement of symptoms, and fear of facing the difficult process of accessing care in public health units^(8)^.

Therefore, one of the relevant obstacles to making thrombolysis feasible is the long pre-hospital delay (period of time between the onset of symptoms or wake up stroke and admission to a health service) in the face of a clinical condition suggestive of stroke. It should be noted that the pre-hospital delay encompasses two main time components. The first is decision time, the period between the onset of symptoms and the decision to seek care, which may be influenced by sociodemographic, clinical, cognitive, emotional, and environmental factors. The second is the transport time (TT), that is, the period between travel and arrival at a health service for the specific treatment. In the second component (TT), the influence of the type of transport used and the possibility of response from the health care network has been observed^(9)^.

In Brazil, the literature remains scarce as to studies on decision time (DT) for people with stroke to seek a health service and on the travel time until admission to a reference service, although the therapeutic window and the in-hospital target times to achieve the benefits of thrombolytic therapy are known. Few studies in the world have specifically portrayed the DT to seek a health service in the face of stroke. Some pointed out the time of arrival at the hospital, varying from hours to days, and mentioned unfavorable socioeconomic status, black race/color, disuse of emergency medical systems, living alone, lack of knowledge about thrombolytic therapy, onset of symptoms at home, personal history of diabetes mellitus, and failure to recognize symptoms and signs as reasons^(10,11)^.

Therefore, it becomes necessary to know the DT for seeking a health service in case of stroke and the factors that influence it, which will show the main predictors for greater pre-hospital delay in the event of a IS and will guide health education provided by nurses. Educational approaches based on the specificities of the clientele allow the achievement of more promising results for the recognition of the clinical manifestations of IS and the correct decision-making regarding the event by the victims and people around them. The nurse’s role in health education is essential, aiming to develop the community’s perception that delaying the decision to seek care in the face of a cerebrovascular event can mean the risk of losing life or permanently limiting it^(12)^.

Based on the aforementioned, the study aimed at verifying the association between sociodemographic, clinical, environmental, cognitive, and emotional factors and the decision time of people with ischemic stroke to seek a health service after the onset of symptoms or wake up stroke.

## METHODS

### Design of Study and Local

Cross-sectional study, carried out in the largest general and public hospital in the North and Northeast Region of Brazil, which has 640 beds, is of high complexity, tertiary, for assistance and teaching, and certified by the Ministry of Health as a Type III Reference Center for the care of people with stroke.

### Sample

Given the gap in the literature on the prevalence of decision time (DT) of people with IS to seek health services, we used the total variability, where we assume that 50% of the population have the characteristic of interest and 50% do not. Adopting 95% confidence and maximum variability of 50%, the sample size resulted in an n (sample) between 196 and 267 participants. The study had a final sample of 304 participants.

Inclusion criteria were medical diagnosis of acute IS recorded in medical records, admission to the study site for treatment, and minimum age of 18 years. As exclusion criteria, symptoms and signs preventing verbal communication in the absence of a companion to answer the research questions, inability prior to the stroke, and more than 10 days after the ictus of the event, considering the possibility of recall bias in the accuracy of the day and time of onset of symptoms.

### Data Collection Instrument

The instrument used in the study is part of the matrix project. It consisted of five parts, with structured, semi-structured, and multiple-choice questions, and was based on the form prepared for a survey on pre-hospital delay in the face of acute myocardial infarction^(9)^.

Part I referred to the participants’ identification information collected from the medical record (name initials and medical record number) and contained questions about sociodemographic data such as age, sex, self-declared race/color, level of education, marital status, employment status, monthly family income, with whom they live and place of residence, which were obtained in the interview.

Part II dealt with questions about clinical variables such as arterial hypertension, diabetes mellitus, smoking, and previous AMI and stroke. Data in Part II were obtained from the medical record and the interview.

Part III was related to the participants’ perception of the severity and origin of the symptoms, to actions when symptoms appear, and to the person who decided to seek a health service. Part IV dealt with the characterization of the environment in which the stroke occurred, including where the symptoms started and who the patient was with when the symptoms started (*wake up stroke*). Data from Parts III and IV were collected during the interview. Part V referred to items for recording the date and time of onset of symptoms and signs or wake up stroke, the date and time of the decision to seek a health service, and the date and time of admission to the hospital at the place of study. Part V data were obtained from the medical record and the interview.

### Data Collection Procedures

Data collection was carried out from March to October 2019.

The data collection team consisted of a doctoral student, a master’s student, and six undergraduate nursing students. The entire team was trained to screen eligible patients at the study site and to collect data. The instrument was pre-tested and its suitability for collecting data to respond to the study objectives was verified.

After agreement to participate in the study, the data collection instrument was applied through interviews. In situations where the participant did not have the clinical, cognitive, and/or emotional conditions to interact with the researcher, the approach was made to his/her companion, as long as he/she was close to the participant and has accurate information about the event.

### Data Analysis and Treatment

Data were a bank in the software Statistical Package for the Social Sciences, version 20.0. Subsequently, they were exported and analyzed using STATA version 12. Categorical variables were analyzed in relative and absolute frequencies. Decision time (DT – time elapsed between the onset of IS symptoms and signs or wake up stroke until the decision to seek a health service) was analyzed as a geometric mean given the asymmetric distribution of the variable.

In the bivariate analyses, to check the association between DT and the independent variables of interest, Robust Linear Regression analysis was used. The variables showing statistical significance at 20% were taken to the multivariate analysis using Robust Linear Regression. Subsequently, the complete model was analyzed with all the variables, using Multiple Linear Regression. The goodness of fit of the model was evaluated using the Akaike Information Criterion (AIC), with the final model with the lowest value being selected. Multicollinearity was evaluated in the model adjustment through the variance inflation factor. The adopted statistical significance was 5%.

### Ethical Aspects

The study was approved by the Ethics Committee on February 21, 2019, with opinion No. 3.159.694. It complies with resolutions 466/2012 and 580/2018 of the National Health Council.

The objectives and importance of the study were explained to the eligible participants, a request was made for their participation, and guidance on signing the Free and Informed Consent Form was provided.

## RESULTS

A sample of 320 people diagnosed with IS was admitted during the data collection period. Of these, 12 had symptoms that prevented communication and were unaccompanied, and four were unable to inform the decision time for seeking a health service after the onset of symptoms or wake up stroke and were unable to answer many of the interview questions. Thus, the analyzed sample consisted of 304 individuals, 179 of which were answered by the participant him/herself and 125 by the companion.

The minimum DT value was 0.02 h (1.5 min) and the maximum 118 h (7,080 min). Given the asymmetry of the DT, it was analyzed in geometric mean (GM), which was 0.30 h (95% CI 0.23–0.39), corresponding to 18 min for the participants to decide to seek care after the onset of IS symptoms or wake up stroke.

As for the sociodemographic characterization of the sample (Table 1), the minimum age was 21 years and the maximum 97 years, *x*= 65 years and SD = 14 years, with most of them being elderly people, since 53.2% were between 60 and 79 years old and 15.5% were elderly women, over 80 years old. The majority lived in Salvador (80.9%), were female (50.7%), self-declared as brown or black (53.5%), had no partner (53.5%), and had a monthly family income of less than three minimum wages (90.2%).

**Table 1.** Association between DT and participants’ sociodemographic variables – Salvador, BA, Brazil, 2021.

Sociodemographic variables	Frequency n (%)	GM decision time in h (CI 95%)	Coef.	Standard error	Value of p*
**Age range in years**					
21 to < 40	19 (6.3)	0.13 (0.04; 0.41)			0.222
40 to < 60	76 (25.0)	0.27 (0.15;0.48)	0.32	0.26	**0.067**
60 to < 80	162 (53.3)	0.37 (0.26;0.54)	0.45	0.25	
≥80	47 (15.5)	0.24 (0.13; 0.48)	0.28	0.27	0.310
**Sex**					
Male	150 (49.3)	0.33 (0.23; 0.50)			0.454
Female	154 (50.7)	0.27 (0.19;0.39)	0.09	0.12	
Self-declared race/color**					
White	49 (16.2)	0.22 (0.11; 0.44)			
Black/brown	254 (83.8)	0.32 (0.24;0.42)	0.15	0.16	0.336
Marital status**					
With a partner	141 (46.5)	0.26 (0.17; 0.38)			
Without a partner	162 (53.5)	0.35 (0.24;0.51)	0.14	0.12	0.255
Level of Education**					
Unfinished high school to higher education	94 (31.3)	0.25 (0.15;0.39)			
Up to elementary school	206 (68.7)	0.32 (0.23; 0.45)	0.12	0.13	0.356
Monthly family income***					
≥ 3 minimum wages	29 (9.8)	0.22 (0.10;0.49)			
<3 minimum wages	267 (90.2)	0.30 (0.23; 0.40)	0.15	0.18	0.418
**City of residence**					
Salvador	244 (80.3)	0.30 (0.22; 0.40)			
Other cities	60 (19.7)	0.32 (0.18;0.54)	0.02	0.14	0.872
**Employment situation**					
With activity	111 (36.5)	0.29 (0.18;0.47)			
Without activity	193 (63.5)	0.31 (0.22;0.43)	0.02	0.13	0.868
**With whom the person lives**					
Does not live alone	245 (80.6)	0.25 (0.19;0.33)			
Lives alone	59 (19.4)	0.66 (0.33;1.33)	0.42	0.16	**0.009**

*p value obtained through linear regression; **n less than 304 due to lack of participant response; ***Minimum wage in 2020: R$1,040.00 = US$192.

In the bivariate analysis, a statistically significant difference was observed at 20% between DT and age group (p = 0.067), with an increase of 14.4 min in DT being observed for those in the age group from 60 to < 80 compared to those aged 21 to < 40. There was also a statistically significant difference between DT and who lived with them (p = 0.009), with an increase of 24.8 min in DT for those who lived alone. There was no association between DT and sex, race-skin color, marital status, level of education, monthly family income, city of residence, and employment status.

As for the clinical characterization (Table 2), 41.1% were smokers or former smokers. A total of 78.6% had systemic arterial hypertension, 28.4% had diabetes, 11.3% had suffered an acute myocardial infarction, and 34.3% had previous strokes. In the bivariate analysis, a statistically significant difference was observed at 20% between DT and diabetes (p = 0.176) and DT and smoking (0.182), noting an increase of 8.4 min in DT for those with diabetes and 13.3 min for smokers. Those with systemic arterial hypertension showed an increase of 462.0 min in DT compared to those without the condition (p = 0.014). There was no association between DT and previous stroke and myocardial infarction.

**Table 2.** Association between DT and clinical variables of study participants – Salvador, BA, Brazil, 2021.

Clinical Variables	Frequency n (%)	GM decision time in h (CI 95%)	Coef.	Standard error	Value of p*
**Arterial hypertension**					
No	65 (21.4)	0.16 (0.09; 0.29)			**0.014**
Yes	239 (78.6)	0.36 (0.27;0.49)	0.35	0.14	
Diabetes**					
No	214 (71.6)	0.25 (0.19;0.35)			
Yes	85 (28.4)	0.40 (0.23;0.70)	0.19	0.14	**0.176**
Prior myocardial infarction**					
No	268 (88.7)	0.30 (0.23;0.40)			
Yes	34 (11.3)	0.31 (0.14;0.71)	0.02	0.18	0.916
**Smoking**					
No	179 (58.9)	0.27 (16)			
Yes	40 (13.2)	0.49 (29.5)	**0.25**	**0.19**	**0.182**
Former smoker	85 (27.9)	0.29 (17.5)	0.01	0.14	0.930
Previous stroke**					
No	199 (65.7)	0.27 (0.20;0.38)			
Yes	104 (34.3)	0.37 (0.23;0.59)	0.13	0.13	0.297

*p value of variables obtained through linear regression; **n less than 304 due to lack of knowledge of the variable by the participant and lack of registration in the medical record.

As for the environmental variables (Table 3), most participants were at home when the symptoms started (79.9%), followed by those who were on the street/public space (10.5%). Most were in the company of someone at the onset of symptoms (78.3%). There was a statistically significant association between the place of onset of symptoms (p = 0.000), with an increase of 11.5 min in the DT being observed for those who were on the street/public space, 15.1 min for those who were at work, and 19.2 min for those at home compared to those already in a health facility when symptoms started. In addition, those who were alone at the onset of symptoms delayed 785.3 min more to make the decision to seek a health service compared to those who were with someone else (p = 0.000).

**Table 3.** Association between DT and environmental, cognitive and emotional variables – Salvador, BA, Brazil, 2021.

Variables	Frequency n (%)	GM decision time in h (CI 95%)	Coef.	Standard error	Value of p*
**Environmental**					
**Site of onset of symptoms**					
Emergency Room or Hospital	8 (2.6)	0.03 (0.01;0.05)			
Street/public space	32 (10.5)	0.22 (0.09;0.53)	0.92	0.22	**0.000**
Work	21 (6.9)	0.28 (0.10;0.81)	1.03	0.24	**0.000**
Home	243 (79.9)	0.35 (0.26;0.47)	1.12	0.13	**0.000**
**Was alone when the symptoms started**					
No	238 (78.3)	0.22 (0.17;0.30)			
Yes	66 (21.7)	0.89 (0.47;1.68)	0.60	0.15	**0.000**
**Cognitive and emotional**					
Judgment of severity of symptoms**					
Severe	190 (64.0)	0.23 (0.17;0.32)			
Not severe	107 (36.0)	0.50 (0.31;0.82)	0.34	0.13	**0.009**
Association of symptoms with stroke**					
Yes	122 (40.3)	0.23 (0.16;0.35)			
No	181 (59.7)	0.37 (0.25;0.53)	0.20	0.12	**0.098**
**Took something to improve**					
No	242 (79.6)	0.24 (0.18;0.33)			
Sim	62 (20.4)	0.69 (0.37;1.30)	0.45	0.15	**0.003**
Waited for symptoms to improve**					
No	226 (74.6)	0.14 (0.11;0.18)			
Yes	77 (25.4)	3.20 (2.00;5.09)	1.37	0.12	**0.000**
**Hid the symptoms**					
No	279 (91.8)	0.27 (0.21;0.36)			
Yes	25 (8.2)	1.07 (0.36;3.13)	0.60	0.23	**0.010**
**Who decided to seek care**					
The victim	76 (25.0)	0.27 (0.16;0.45)			
Another person	228 (75.0)	0.31 (0.23;0.43)	0.07	0.13	0.613

*p value obtained through linear regression; **n less than 304 due to lack of information about the variable.

Regarding the cognitive and emotional variables (Table 3), most considered the symptoms to be severe (66.0%), did not associate them with the IS (59.7%), did not take something to improve (79.6%), did not wait to see if the symptoms would improve (74.6%), and did not hide the symptoms (91.8%). The decision to seek a health service was predominantly taken by a person close to the victim (75.0%).

In the bivariate analysis, those who did not consider their symptoms severe (p = 0.009), took something to improve (p = 0.003), waited their symptoms to improve (p = 0.000), and hid their symptoms (p = 0.010) showed an increase in DT, respectively of 16.3; 26.9; and 47.9 minutes. There was a 20% statistical difference between DT and recognition of stroke symptoms (p = 0.098), noting that people who did not recognize it increased the time to decide to seek a health service by 8 min. There was no association between DT and who decided to seek the health service.

The variables showing statistically significant difference in the bivariate analyses (p ≤ 0.20) were taken to the multivariate analysis presented in the linear regression models (Table 4).

**Table 4.** Robust linear regression models stratified by blocks of clinical (Model A), cognitive and emotional (Model B), environmental (Model C), sociodemographic (Model D) variables, complete linear regression model (Model E), and final model (Model F) – Salvador, BA, Brazil, 2021.

Variables	Model A (CI 95%)	Model B (CI 95%)	Model C (CI 95%)	Model D (CI 95%)	Model E (CI 95%)	Model F (CI 95%)
**Clinical**						
Hypertension						
No						
Yes	0.33 (0.04;0.62)				0.18 (–0.89;0.45)	0,24 (0,01;0,47)
Diabetes						
No						
Yes	0.17 (–0.13;0.47)				0.08 (–0.15;0.31)	
Smoking						
No						
Yes	–0.02 (–0.15;0.12)				0.06 (–0.06;0.17)	
**Cognitive and emotional**						
Symptoms associated with stroke						
No						
Yes		–0.00 (–0.20;0.20)			–0.01 (–0.22;0.19)	
Took something to improve						
No						
Yes		0.06 (–0.18;0.30)			0.09 (–0.14;0.33)	
Waited for symptoms to improve						
No						
Yes		1.33 (1.07;1.59)			1.26 (1.00;1.51)	1.27 (1.04;1.50)
Hid the symptoms						
No						
Yes		0.08 (–0.31;0.48)			0.04 (–0.33;0.41)	
Symptoms severity						
No						
Yes		–0.05 (–0.27;0.18)			–0.01 (–0.22;0.21)	
**Environmental**						
With whom the person was at the onset of symptoms						
Wasn’t alone						
Was alone at onset of symptoms			0.66 (0.35;0.97)		0.50 (0.18;0.82)	0.53 (0.26;0.80)
Where the person was at the onset of symptoms						
Emergency Unit (UPA)/Hospital						
Street/public space			0.87 (0.41;1.33)		0.58 (–0.03;1.18)	0.65 (0.04;1.26)
Work			0.83 (0.37;1.30)		0.75 (1.19;1.32)	0.79 (0.19;1.38)
Home			1.04 (0.73;1.35)		0.79 (0.26;1.29)	0.87 (0.33;1.40)
**Sociodemographic**						
Age range						
21 to < 40 years						
40 to < 60 years				0.37 (–0.15;0.88)	0.03 (–0.35;0.42)	
60 to < 80 years				0.47 (–0.01;0.95)	0.14 (–0.26;0.54)	
> 80 years				0.23 (–0.31;0.76)	0.12 (–0.33;0.59)	
With whom the person lives						
Does not live alone						
Lives alone				0.44 (0.11;0.77)	0.98 (–0.22;0.42)	
**AIC**	812.37	710.74	791.56	809.007	693.40	677.76
**R2**	4.5%	10.3%	33.1	3.0%	42.0%	41.0%

In Model D, sociodemographic block, it was observed that living alone increased the DT for seeking a health service by 10.3 min after the onset of symptoms adjusted for age.

In Model C, environmental block, it was noted that being alone at the time of symptom onset increased DT by 4.6 min. It was also observed that the participants who were on the street/public space, at work and at home showed, respectively, an increase in DT of 8.3; 7.5 and 12.9 min when compared to those who were admitted to a hospital or an emergency care unit.

In Model B, cognitive and emotional block, those who waited for the improvement of symptoms delayed an additional 158 min to make the decision to seek a health service after the onset of symptoms compared to those who did not wait, with the model being adjusted through the variables association of IS symptoms, hiding symptoms, and judgment of symptom severity.

In Model A, clinical block, hypertensive participants had a 9.8-minute increase in DT compared to those without hypertension, adjusted for diabetes and smoking.

In Model E, the complete model, with all variables analyzed concomitantly, it was noted that participants who waited for the improvement of symptoms, who were alone at the onset of symptoms, who were at work and at home compared to those who were in an emergency room or hospital had a respective increase of 0.28; 0.11, and 0.08 minutes on DT.

However, in the final Model F, the one with the lowest AIC, whose coefficient of determination (R2) explained the variables associated with DT by 41%, showed an increase in DT for participants with arterial hypertension (0.5 min), who waited for the improvement of symptoms (10.8 min), were alone at the onset of symptoms (1.4 min), were on the street/public space (2.1 min), at work (3.2 min), and at home (3.9 min).

There was no multicollinearity between the variables analyzed, in all models.

## DISCUSSION

This study showed that IS mostly affected elderly people, without a partner, black or brown, with a monthly family income of less than three minimum wages, and affected males and females in identical proportions, data that are similar to other Brazilian^(13,14)^ and international^(6,15)^ studies.

Regarding the association of DT with sociodemographic factors, the bivariate analysis showed a greater delay in the decision to seek a health service for people over 40 years of age, with borderline statistical significance for those in the age group from 60 to less than 80 years. International studies, although they have explored the relationship between time of arrival at a health service and sociodemographic variables, have also identified a greater delay for people in older age groups, such as between 55 to 64 years and 65 to 74 years^(6)^, above 80 years^(16)^

In this study, the delay in the decision to seek a health service was significant for people who lived alone and were single, which may be related to the lack of anyone around to witness the occurrence of the event^(10.15)^.

Although the variables sex and DT are independent, a greater DT was observed for men, unlike international research that found a longer time of presentation to a health service for women^(15)^, justified by the greater probability of them having nonspecific symptoms such as chest pain, difficulty breathing, concentration and memory problems, nausea, vomiting, feelings of irritability, and restlessness when compared to men^(7)^. The finding that men took longer to decide to seek care may be associated with the culture of masculinity, in which they are considered invulnerable, strong and virile, imposing a posture of power that does not allow fragilities to appear. Masculinity models can distance men from self-care and the search for health services, despite occupying an important position in the profiles of morbidity and mortality from various causes^(16)^.

In this investigation, there was no statistically significant relationship between DT and variables that express a situation of social vulnerability, such as black race/color, lower education and income, and inactive work status, although participants in these classes had a higher DT. Other investigations observed the same vulnerability indicators as risk factors for delay in presenting to a health service, such as low income, black race^(17,18)^ and the unemployment situation^(15)^. Precarious access to health services in Brazil by the black population may be based on structural racism, materialized in health institutions and on the lack of implementation of policies aimed at this population^(19)^.

In a study carried out in Columbia, a lower probability of presenting to a health service within three hours of the onset of symptoms was observed for black people. Furthermore, black people who arrived within the first three hours were almost half as likely to undergo thrombolysis compared to white people, although the rate of treatment for those eligible was similar for blacks and whites. Effective interventions aimed at scaling up treatment in this population have to focus on culturally designed education programs to address specific barriers and on public policies and actions that ensure equity, comprehensiveness, and universality of health care^(17)^.

As for the clinical variables, a predominance of participants with systemic arterial hypertension (SAH) was observed, which reinforces it as the main risk factor for cerebrovascular diseases^(20)^. In addition, other clinical data showed risk factors for stroke, as almost half of the sample was a smoker or former smoker and about a third had diabetes. Among these variables, SAH was significantly associated with greater DT, but a greater DT was also found, with no statistical difference, for people with diabetes, smokers or ex-smokers. Systemic arterial hypertension^(21)^ and diabetes^(15,22)^ have already been pointed out as a delay factor in the presentation to health services. Diabetes has been associated with late presentation^(15,22)^, possibly because the sensory and autonomic dysfunction related to the disease can confuse victims or people around them as to the nature of the symptoms, whether typical of diabetes or related to a new event. DT and previous acute myocardial infarction or stroke were independent, but a higher DT was observed for participants with previous stroke, corroborating the investigation carried out in Singapore^(21)^. People who have already had a stroke may have deficits that make it difficult to judge new symptoms, mobility difficulties, and are therefore more prone to delay in making the decision to seek a health service.

Smoking is an important risk factor for the occurrence of stroke, with negative consequences on the lives of survivors, as they have worse functional results compared to non-smokers, three months after the stroke^(23)^. It is necessary to face it, given that it is the only totally preventable risk factor for cardiovascular disease and death^(24)^. The proportion of smokers and former smokers was high in this study and, in the bivariate analysis, smokers had a higher DT, despite the lack of statistical significance. People who smoke may face barriers, embarrassment, and discrimination when entering health services, as they are initially susceptible to a professional approach focused on combating the smoking habit, to the detriment of greater visibility of their health needs, which can contribute to the delay in seeking these services.

With regard to environmental variables, in the bivariate analyses, a greater DT was observed for people who were alone at the onset of symptoms, corroborating a study carried out in Saudi Arabia^(25)^ and strengthening other findings of the present investigation in which the delay in the decision to seek a health service was significant for people who lived alone and for single people. Thus, not being accompanied during the manifestation of the event can influence a greater DT.

Moreover, in the environmental block, a greater DT was also identified for people who were at home, at work, and on the street/public space compared to hospitalized ones. It was to be expected that hospitalized people would ask for immediate help from the health team in the face of the appearance of new symptoms and signs, due to the ease of access to professionals. It should also be noted that the greater DT for those who were at home, on the street/public space or at work, when each of these categories was compared to the reference category, may be related to the fact that in public space people have others in their surroundings whom they can consult and ask for help and, thus, decide more promptly to seek care. As noted, those who lived alone took longer to call for help.

As for cognitive and emotional factors, bivariate analyses showed that not considering the symptoms and signs of severe stroke led to greater DT, corroborating findings of the study carried out in Singapore^(26)^ and indicating the participants’ understanding that emergency care was not necessary. People who recognized the event as a stroke decided earlier to seek help, corroborating the study carried out in Saudi Arabia^(25)^ and possibly evidencing the perception of the seriousness of the clinical situation.

Taking something to improve and waiting to see its improvement fueled the mistaken hope that the clinical condition could be cured or minimized with one’s own resources. These participants’ attitudes consist of actions that need to be fought, as they led to greater DT. Those who hid the symptoms also had higher DT and this attitude can highlight the lack of knowledge about the seriousness of the event, as well as the fact that the victims did not want to admit that something extraordinary was happening or worry the people around them.

The fact that a third of the sample did not consider the symptoms and signs of stroke to be serious, that more than half did not associate them with a cerebrovascular event, that a quarter took something to get better, and a quarter believed that the symptoms would improve, reinforces the importance of educational programs aimed at the recognition of stroke by the population and the correct action in face of it. These results corroborate a study carried out in Salvador that showed an association between the recognition of stroke symptoms and early arrival at the hospital^(27)^.

No statistically significant difference was observed between DT and who decided to seek care in view of the occurrence of the ischemic stroke. However, the fact that the decision-making agent was more frequently not the victim him/herself, but someone around him/her was highlighted. This may be associated with the fact that the event causes cognition deficits, inability to articulate speech, decreased strength, lowered level of consciousness, among other factors. This finding also reinforces the importance of health education aimed at the general population to acknowledge and act in case of stroke.

When the variables associated with DT with statistical significance at 20% in the bivariate analyses were taken to the multiple analysis, it was confirmed, in the more parsimonious multivariate model, that variables from several blocks remained associated with greater DT, reinforcing the influence of clinical, environmental, and cognitive and emotional factors in the delay in the decision to seek care in case of IS and stressing the need for population education actions and public policies for social inclusion in access to health services. Variables such as SAH, waiting for symptoms to improve, being unaccompanied, on the street/public space, at work or at home at the time of the event contributed to greater DT.

The greater DT for hypertensive participants, combined with the greater risk of suffering an IS, are facts that make them the target audience of priority attention for educational interventions that promote emphasis on the control of this risk factor, the identification of symptoms and signs of stroke, and the need for quick presentation to a specialized center for treatment, soon after the onset of symptoms.

The attitude of taking something to improve and waiting for the symptoms to improve, predictive of greater DT, was opposed to the recommendation that emergency medical services should be immediately called by the victim, as they provide agility in care, favoring the use of thrombolytic therapy at golden hour^(4)^. Feeding the hope of improvement of symptoms and making attempts to minimize them may also be associated with the fear of coping with difficult access to health services, aggravated by high demand and low supply. In Salvador, a city with an estimated population of 2,900,319 inhabitants (last IBGE census 2010 total of 2,675,656 people) has only one stroke unit with 14 beds. Studies have shown that the annual occurrence of stroke in Brazil is 108 cases per 100,000 inhabitants^(2)^. Fallacies and negative experiences also make the Brazilian Public Health Service - SUS discredited in its ability to solve problems. Therefore, it is essential to focus attention on improving the credibility of SUS services, on increasing the supply of specialized services, and on the knowledge of victims and the general population about which health service to call in the presence of an IS in its territory of occurrence.

It should also be noted that the location where the study participants were at the onset of symptoms significantly contributed to greater DT. The noted occurrence of IS in people who were already hospitalized draws attention to the need for guidance and training for teams that do not routinely deal with people with stroke or are unaware of the possibilities of intervention in the acute phase of stroke. Greater delay time to decide for those who were at home, followed by the ones on streets/public spaces and at work, reiterates the importance of training the population to recognize the event. Being at home was also identified as a delay in presenting to a health service^(28)^, which may be related to the fact that the home environment is considered more conducive to accommodating symptoms, resting, or waiting for a decision by a third party.

The findings of this study on factors associated with longer DT, although not providing data that allow generalization, advance in identifying the variables that influence it, contributing to the identification of risk groups for greater pre-hospital delay in the face of stroke. The results obtained have to be considered in health education programs and emphasize the importance of public policies and management of health services aimed at health education for professionals, patients, family members, and the general population, targeting the recognition of symptoms and signs of a cerebrovascular event, the appropriate action when facing it to reduce sequelae and death from stroke.

The limitations of this study include the fact that data collection was carried out in a single, public hospital in the state of Bahia, which is a reference for the care of people with ischemic stroke and may keep specific characteristics of the sample, and the fact that the main variable is time reported by the participants, which may present recall bias.

The main contribution of this study is to be the first to specifically investigate decision time and its association with variables of interest, something unprecedented in the Brazilian literature.

## CONCLUSIONS

Participants delayed an average of 18 min to decide to seek care at a health service and the DT was influenced by variables of clinical (HAS), environmental (being alone or in an environment other than a health service), and cognitive and emotional (wait for the symptoms to improve) nature.

The identification of factors involved in the DT for pre- hospital delay in the face of ischemic stroke provides subsidies for the development of educational actions so that one can succeed in reducing this time, optimizing the results of time-dependent therapies.

Health education activities aimed at people with risk factors for stroke and the community in general should consider the variables that contributed to greater DT.

The nurse, understanding the influence of the factors involved in the decision time to seek a health service, can act to help people to increase their decision-making skills about their own health and that of individuals around them actively and effectively, understanding the importance and valuing every minute for the seek a health service in case of the onset of IS symptoms or wake-up stroke.
